# Eating Disorders, Co‐Morbid Disorders and Early Risk Factors Amongst Post‐9/11 Veteran Men and Women

**DOI:** 10.1002/jclp.23756

**Published:** 2024-12-08

**Authors:** Zafra Cooper, Brian N. Smith, Shannon Kehle‐Forbes, Julianne Dorset, Karen S. Mitchell

**Affiliations:** ^1^ Department of Psychiatry Yale University School of Medicine New Haven Connecticut USA; ^2^ National Center for PTSD at VA Boston Healthcare System Boston Massachusetts USA; ^3^ Chobanian & Avedisian School of Medicine Boston University Boston Massachusetts USA; ^4^ Minneapolis VA Healthcare System Minneapolis Minnesota USA; ^5^ Department of Medicine University of Minnesota Minneapolis Minnesota USA; ^6^ Department of Social Work Yale New Haven Hospital New Haven Connecticut USA

**Keywords:** comorbid psychiatric disorder, early risk factors, eating disorders, treatment needs, veterans

## Abstract

**Objective:**

To assess, by interview, the rates of eating disorders in a nationally representative sample of recent veterans, describe their DSM‐5 eating disorder diagnoses and the occurrence of comorbid psychiatric disorders. To conduct an exploratory case‐control analysis of previously documented and additional specific military risk factors before eating disorder onset to inform studies of prospective risk.

**Method:**

Using a two‐stage design, probable cases and controls were identified by screening questionnaires from a sample of 1494 veterans who completed a survey study and interviewed to establish case status and confirm probable co‐morbid psychiatric diagnoses. Previously documented risk factors and military risk factors occurring before disorder onset were investigated.

**Results:**

Ninety‐one cases and 51 controls were confirmed. Weighted prevalence for any eating disorder was 5.2%, with estimates for anorexia nervosa, bulimia nervosa, binge eating disorder and other specified eating disorder being 0.01%, 0.6%, 1.4%, and 1.6%, respectively. Seventy‐nine (86.8%) confirmed cases had a diagnosis of one or more co‐morbid psychiatric disorders. Previously documented risk factors were associated with subsequent case status, while in this sample, military risk factors were not.

**Discussion:**

Rates of eating disorder and co‐occurring psychiatric disorders in recent veterans were comparable to those reported for non‐veterans, with levels of posttraumatic stress disorder likely higher. As co‐occurring psychiatric disorders, particularly posttraumatic stress disorder, may complicate achieving good outcomes with existing evidence‐based treatments, there is an urgent need to adapt them where necessary to improve outcomes. Military risk factors may maintain or exacerbate pre‐existing problems and need to be investigated alongside other maintaining factors in longitudinal studies.

**Public Significance:**

Rates of eating disorder and co‐occurring psychiatric disorders in recent veterans were comparable to those reported for non‐veterans, highlighting a need to detect eating problems and address unmet treatment need. Co‐occurring psychiatric disorders may complicate achieving good outcomes with existing treatments, emphasising a need to adapt them to improve outcomes. Investigating maintaining factors, including military factors in longitudinal studies will likely aid treatment development.

Eating disorders (EDs) are debilitating disorders distributed broadly across the population, occurring across sex, race/ethnicity, age and education levels (Udo and Grilo 2018). The three specifically designated EDs (anorexia nervosa (AN), bulimia nervosa (BN) and binge eating disorder (BED)) and their variants, recognized by the fifth edition of the *Diagnostic and Statistical Manual of Mental Disorders* (American Psychiatric Association 2013), are associated with significantly elevated rates of other psychiatric disorders, physical health conditions and significant impairment in quality of life (Udo and Grilo 2018, 2022). EDs are also associated with increased morbidity and mortality (Keski‐Rahkonen 2021; Van Eeden, Van Hoeken, and Hoek 2021) and substantial social and economic costs (Streatfeild et al. 2021). These disorders can be difficult to treat, with longer duration of disorder predicting worse outcome (Vall and Wade 2015). Despite the existence of evidence‐supported specialist interventions for EDs (Cooper and Bailey‐Straebler 2015; Kazdin, Fitzsimmons‐Craft, and Wilfley 2017) there is a well‐documented unmet need for treatment (Hart et al. 2011; Striegel Weissman and Rosselli 2017).

EDs and subthreshold disordered eating in United States (US) military veterans have received relatively little research attention until recently (Masheb et al. 2021; Mitchell et al. 2021). This is, despite evidence that veterans have been found to have higher rates of mental health problems as compared with the general public (Seal et al. 2009) and that EDs occur in the context of other psychiatric disorders (Striegel Weissman and Rosselli 2017). While higher rates of EDs amongst veterans as opposed to non‐veterans have been suggested (Cuthbert et al. 2020), studies have produced a wide range of estimates, likely related to the varying methods used to identify these disorders. In studies using electronic medical records from the Veterans Health Administration (VHA), one‐ and 5‐year prevalence for any eating disorder among post‐9/11 veterans was estimated as 0.1% and 0.2%, respectively (Blais et al. 2017; Livingston et al. 2019). As there is no routine screening for EDs in the VHA and these disorders are usually under‐reported, prevalence estimates from medical records are likely to be underestimates. Studies using self‐report measures have produced much higher rates. A study of post‐9/11 veterans receiving healthcare from the VHA in two US states found that 18.6% of women and 7.9% of men self‐reported disordered eating (Slane et al. 2016). Another self‐report study of a nationally representative sample of US veterans produced similar rates; 18.6% of women and 8.5% of men were estimated to have a probable ED (Mitchell et al. 2021). A further large study of those enrolled in VHA health care estimated that 32.8% of women and 18.8% of men had any probable DSM‐5 eating disorder (Masheb et al. 2021), while a self‐report survey of a nationally representative sample of recently separated veterans found that 7.9% met probable criteria for any eating disorder (Mitchell et al. 2022).

Consistent with the general literature on ED prevalence (Lindvall Dahlgren and Wisting 2016), studies of veterans that have used interviews to assess the presence of an eating disorder have reported lower estimates. Three studies of veterans within the VHA have reported prevalence estimates of DSM‐IV EDs ranging between 3% and 4.7% when assessed by interview (Curry et al. 2014; Litwack et al. 2014). While these estimates are comparable to those reported for the US population in general (Galmiche et al. 2019), none of these veteran samples were nationally representative.

Earlier work reporting elevated associations of EDs with other mental health conditions in veterans involved the study of EDs in populations identified as having another condition. Examples of groups studied are those with post‐traumatic stress disorder (PTSD) or trauma exposure, especially military sexual trauma (MST), depression, anxiety or substance use (Breland et al. 2018; Hoerster et al. 2015; Litwack et al. 2014; Maguen et al. 2012). Recent studies focusing primarily on veterans with EDs or disordered eating have confirmed elevated rates of comorbid disorders or symptoms in these groups. Rates of PTSD, major depressive disorder and alcohol use, as assessed by medical records, were notably high among those with self‐reported disordered eating (Slane et al. 2016). Symptoms of stress, anxiety, trauma, depression and insomnia were more common in those with a probable ED assessed by self‐report than those without an ED. (Masheb et al. 2021). Increased rates of trauma exposure, especially MST, PTSD and depression have been cited as possible explanations or risk factors for the elevated rates of EDs amongst veterans (Cuthbert et al. 2020) as have strict military weight and fitness requirements, and the high stress military environment (Cuthbert et al. 2020). To date, there has been no nationally representative study of veterans that has reported on ED diagnoses, disordered eating and accompanying psychiatric disorders evaluated by interview. Nor have there been studies investigating whether previously established general risk factors for EDs (conduct disorder, family psychiatric disorder, bullying, physical and sexual abuse) and ED specific risk factors (individual and family history of obesity, and high parental demands) (Hilbert et al. 2014; Striegel‐Moore et al. 2002; Striegel‐Moore et al. 2005) are true risk factors in this population (Jacobi et al. 2004), that is, that they pre‐date the onset of disorder. Likewise, the role of military factors (MST, pressure to conform to weight and fitness standards and the high stress military environment) associated with eating disorders in cross sectional studies (Arditte Hall et al. 2017, 2018), have not been investigated pre‐onset.

The present study sought to address these gaps by using gold standard interviews to assess a subsample of veterans drawn from a nationally representative study of recently separated US military veterans who had initially been recruited to take part in a self‐report survey of eating behaviors, military experiences and healthcare use (Mitchell et al. 2022) In this subsample we aimed to investigate the rates of EDs in both men and women; report the occurrence of the different DSM‐5 eating disorder diagnoses and examine the occurrence of comorbid psychiatric disorders. In an exploratory case‐control analysis, we investigated previously researched and identified general and specific risk factors together with the military specific risk factors, hypothesized to contribute to EDs in veterans, but not previously investigated in this way. The objective of the case‐control analysis was to generate hypotheses concerning risk factors that might warrant further investigation and inform more robust longitudinal studies able to assess prospective risk.

## 1 Method

### 1.1 Participants and Procedure

*Initial survey study recruitment:* Initial recruitment of participants for the self‐report survey, details of the sample and survey findings have been described in greater detail elsewhere (Mitchell et al. 2022). Briefly, a research firm was used to recruit and administer the survey. Potential participants (*N* = 7700) who had separated from the service within the past 18 months were selected from a database of US military service members and veterans using the VA/DoD identity Repository (VADIR). Women were oversampled to achieve a 1:1 ratio of women to men. Of the 7687 with locatable mailing addresses, 1494 (19.4%) surveys were completed covering eating disorder symptoms, mental health, healthcare use and attitudinal and logistical barriers to care. The vast majority were completed online (1249/1494), with the remainder completed on paper, between February 2020 (before the major shutdowns due to the COVID‐19 pandemic) and May 2020 (during and after the shutdowns). Participants indicated whether they would be willing to be contacted, if selected, for a subsequent research interview.

*Recruitment for interview study:* Of those who agreed to further contact, the survey research firm and KSM identified the contact details for 200 veterans with a probable ED and 193 of those who had neither a probable ED nor a probable other psychiatric disorder to serve as potential age and sex matched controls for the exploratory case control part of the study. Participants who endorsed specific behaviors required for a possible ED diagnosis on the Eating Disorder Diagnostic Scale (Stice, Telch, and Rizvi 2000) or the Eating Disorder Examination‐Questionnaire (Fairburn and Beglin 2008) and achieved a score ≥ 16 on the Clinical Impairment Assessment (Bohn et al. 2008) as well as a sample of participants who did not report any clinically significant symptoms of EDs, depression, anxiety, PTSD, or substance use were selected. Scoring algorithms are described and justified in the Table S1.

Research staff contacted participants by telephone, or by mail if no phone number was available, to invite them to take part in the follow up interview study. Interested participants were provided with a link to the study website and a study information sheet and were able to consent to the study, opt out from further contact or request a telephone call for further clarification. Contact was attempted by telephone, text message or mail on up to three occasions. After consent, diagnostic interviews were arranged to determine ED status and to confirm the presence of other psychiatric disorder for those whose ED case status was established and whose self‐report scores indicated that they exceeded clinical cut offs for psychiatric disorder (see Table S1). Of the 200 probable cases, it was possible to contact 147 (73.5%); 16 refused to participate, 131 consented to interview and a further 12 were not contactable after consent. The EDE diagnostic interview was completed by 119 (60%) probable cases. From the pool of potential controls (*n* = 193), it was only possible to contact 87 (45%); 24 refused to participate, 63 consented, 3 were lost to subsequent contact, and 60 completed the diagnostic interview.

**Figure 1:**
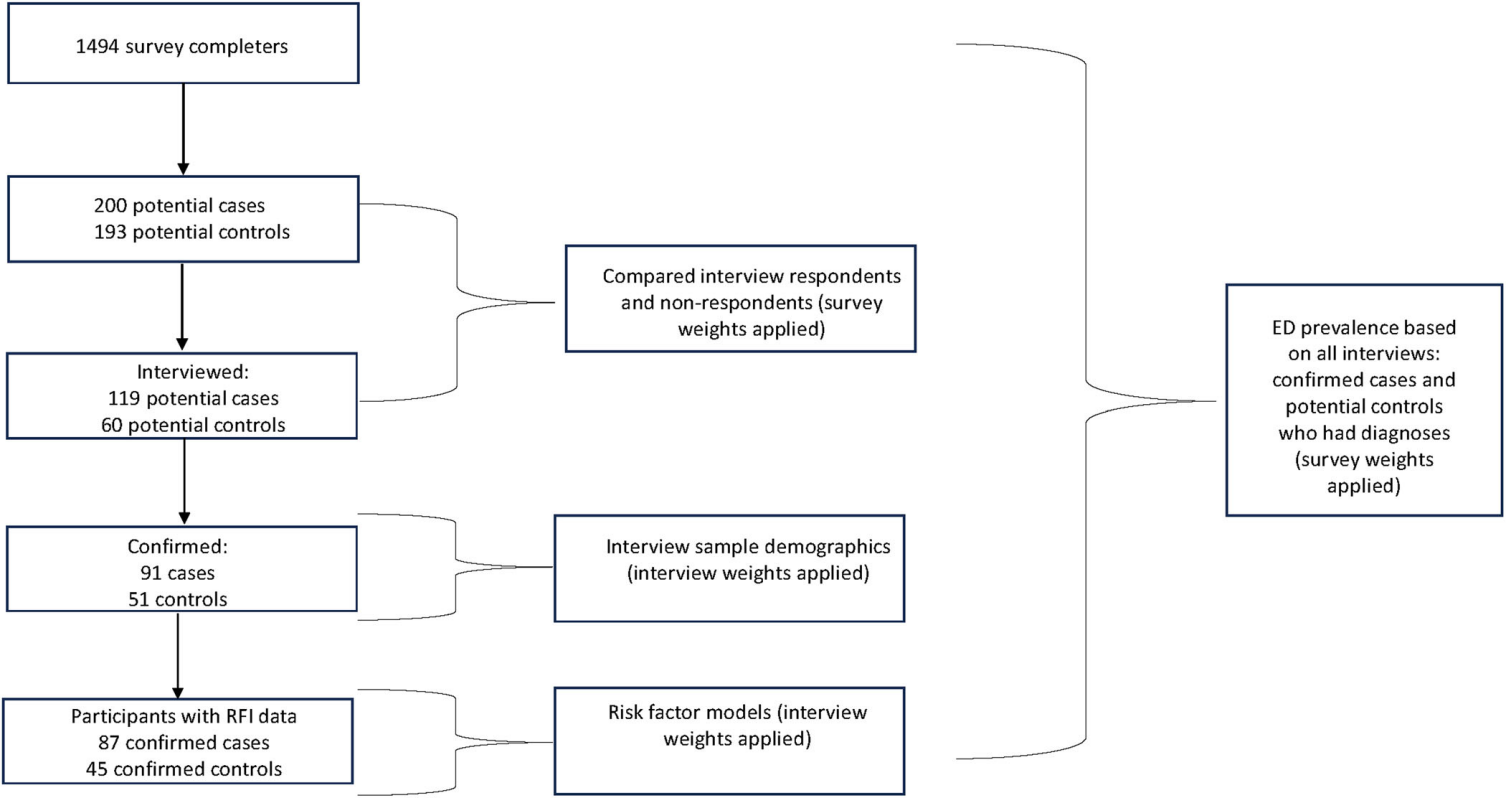
Flow chart of participants identified and interviewed.

Of the 119 probable cases, 92 were confirmed as having an ED at interview. Of the 60 potential controls, 51 were confirmed at interview as not having an ED. To investigate risk factors that occurred before the onset of disorder, confirmed cases and their matched controls took part in a further interview assessing a range of potential risk factors pre‐dating index age (see below). Controls were assigned the index age of the one participant with whom they were matched. One of the 92 confirmed cases did not receive a sample weight due to an error and was excluded from analyses. Four confirmed cases did not complete the risk factor interview. Of the 51 confirmed controls, 47 were potential matches for the confirmed cases, but two did not complete the risk factor interview. See Figure 1 for a flow chart of participants identified and interviewed.

Recruitment for the interviews began in August 2020 and ended in January 2022. Participants were interviewed by voice or video call using a HIPAA compliant method and all data were stored via REDCap. Interviews were audio recorded to monitor their quality provided participants consented. Refusing permission (19 of 179 interviewed; 10.6%) did not disqualify a participant. Participants were compensated, receiving between $90–$150 depending on the number of interviews they completed. All study procedures for the interview portion of the study were approved by Yale University Institutional Review Board (IRB); the VA Boston Healthcare System IRB approved the survey portion of the study.

Interviews were conducted by three research staff trained by ZC. Training involved attending a workshop and watching role play interviews. Interviewers completed mock interviews and received feedback on their audio recordings. During the study, the team met twice weekly via videoconferencing to discuss ratings and diagnostic decisions for each interview. In addition, ZC listened to audio recordings of 15% of the recorded interviews to monitor quality.

### 1.2 Diagnostic Interviews

*Eating Disorders:* The Eating Disorder Examination (EDE) is an investigator‐based interview of eating disorder psychopathology (Cooper and Fairburn 1987). The current version of the EDE (17.0D) (Fairburn, Cooper, and O'Connor n.d.) was used to confirm the diagnostic status of those who screened positive for ED and to establish the age of onset of the first clinically significant ED symptom (“index age”‐see below). The newly included DSM‐5 disorders (Avoidant Restrictive Food Intake Disorder (ARFID), Rumination Disorder and Pica) were not assessed.

*Psychiatric comorbidity:* The Structured Clinical Interview for DSM‐5 (SCID‐5) (First et al. 2015) is an investigator‐based interview for the assessment of mental health disorders. Diagnoses were established among the ED cases using the following modules: Mood Disorder, Substance‐Use Disorder, Anxiety Disorder, Obsessive‐Compulsive, and Trauma.

### 1.3 Risk Factors

The occurrence of risk factors before the onset of the ED was assessed using a modified version of the Oxford Risk Factor Interview (RFI) (Fairburn, 1997). It covers seven broadly defined risk domains (parenting quality, family eating and weight concerns, family and individual health, family mental health, individual mental health physical and sexual abuse and other adverse environmental circumstances) including the occurrence of a range of life events in the 12 months before index age. The RFI focuses on the period before the onset of clinically significant eating symptoms, defined as index age, to ensure that exposure preceded the onset of disorder. It was modified to assess military‐specific risk factors before onset of disorder, adding items to assess combat exposure and MST from the Deployment Risk and Resilience Inventory‐2 (DRRI‐2)(Vogt et al. 2013). Difficulty in conforming to military weight/fitness standards was assessed using items similar to those in the Millennium Cohort assessment (Jacobson et al. 2009) and previous studies (Garber et al. 2008). Exposure to a risk factor was rated on a 5‐point scale ranging from 0 = no exposure to 4 = high severity, long duration, or high frequency of exposure. To reduce the likelihood of false positives, data were recoded into 0 = no definite exposure (initially coded 0, 1, or 2) versus 1 = definite exposure (initially coded 3 or 4) (Hilbert et al. 2014).

### 1.4 Demographic Information

Participants self‐reported their gender, race, education level, sexual orientation and ethnicity.

#### 1.4.1 Statistical Analyses

As previously described (Mitchell et al. 2022), sample weights were applied to analyses to calculate more accurate standard errors and increase representativeness of findings to the population of veterans who separated from service within the 18 months before the survey. The *initial weight* for the interview subsample was the final weight computed for the survey respondents. This weight was adjusted for nonresponse and raked (iterative poststratification) such that the final weighted sample of 1494 respondents to the DoD Military Service and Health Survey matches the distribution of the study population for the 5‐year age category, sex, race, and ethnicity. The second step adjusted the initial weights for nonresponse by computing nonresponse adjustment factors within weighting cells. Weighting cells were defined by variables known for both respondents and nonrespondents, from the VADIR database and the survey. Nonrespondents included veterans who did not agree to participate in follow up studies, those who refused to participate in the interview, and those who were not contactable for interview. The *adjusted initial weight* for respondents is the initial weight multiplied by the corresponding adjustment factor, whereas the adjusted initial weight for nonrespondents is zero. The final step computes adjustments to ensure the weighted respondents correspond to expected proportions from the sampling frame. The *poststratified adjusted weights* for respondents are the nonresponse adjusted weight multiplied by the corresponding poststratification adjustment factor and are the final weights used in analyses for the interview subsample. The interview sample weight was applied to risk factor analyses and descriptive analyses for the interview subsample to ensure accuracy of the standard errors (see Figure 1).

The “survey” package in R 4.2.1 was used to compare interview respondents and non‐respondents and to calculate descriptive statistics for the analytic sample (confirmed cases and controls). We calculated prevalence estimates of EDs in the total sample of 1494 participants, as the survey data weight was calculated to provide estimates that are representative of post‐9/11 veterans. The estimates account for missing diagnostic data (see Figure 1) for those who did not complete an interview.

Conditional logistic regression models, with sample weights applied, were estimated using SAS PROC SURVEYLOGISTIC Given the small numbers of case and control participants, each potential risk factor was included in a separate model for these exploratory analyses. Potential risk factors were coded as present or absent, except for the number of adverse life events, which was a sum score. The outcome variable in these models was defined as case (1) or control (0). Models were adjusted for age and gender and methods allowed for unequal group sizes. Two individuals, identified as nonbinary and included in the descriptive statistics, were excluded from the models that controlled for gender as the inclusion of a category with only two participants resulted in model estimation problems.

## 2 Results

### 2.1 Description of the Confirmed Cases and Controls

Table 1 provides demographic information for the 91 confirmed cases and the 51 confirmed controls.

**Table 1:** Demographic information for confirmed case and control participants.

	Cases M (SD)	Controls M (SD)	Total M (SD)
	*N* = 91	*N* = 51	*N* = 142
Age at time of survey	27.9 (6.7)	29.1 (5.4)	28.7 (5.8)
Index age	17.2 (5.4)	—	—
Body mass index	30.0 (5.5)	26.2 (4.0)	27.4 (4.8)
	Cases *n* (%)	Controls *n* (%)	Total *n* (%)
*Gender*			
Men	27 (60.9)	29 (87.0)	56 (78.8)
Women	62 (37.2)	22 (13.0)	84 (20.6)
Transmale	1 (0.6)	0 (0.0)	1 (0.2)
Genderqueer	1 (1.2)	0 (0.0)	1 (0.4)
*Sexual orientation*			
Heterosexual or straight	74 (87.1)	45 (95.8)	119 (93.4)
Gay or lesbian	2 (0.02)	4 (0.03)	6 (0.03)
Bisexual	15 (0.1)	2 (0.01)	17 (0.04)
*Education*			
High school or GED	25 (41.1)	6 (18.4)	31 (25.6)
Some college or associate degree	43 (42.8)	17 (32.2)	60 (35.6)
Graduated from a 4‐year college	16 (12.1)	18 (33.6)	34 (26.9)
Completed a postbaccalaureate degree	7 (4.0)	10 (15.7)	17 (12.0)
*Race*			
Pacific Islander or Native Hawaiian	0 (0.0)	0 (0.0)	0 (0.0)
American Indian or Alaska Native	3 (2.2)	0 (0.0)	3 (0.7)
Black	13 (14.7)	6 (11.9)	19 (12.8)
White	71 (79.0)	43 (85.2)	114 (83.0)
Asian	6 (6.9)	0 (0.0)	6 (2.2)
Other race	4 (2.9)	1 (0.6)	5 (1.3)
*Ethnicity*			
Hispanic/Latinx	19 (16.5)	9 (8.8)	28 (11.2)
Non‐Hispanic/Latinx	72 (83.4)	42 (91.2)	114 (88.8)

*Note:* Raw frequencies and weighted means and percentages are reported. Sample weights account for the oversampling of women. The higher number of women in the group of cases is reflected by the raw frequency. However, the weighted percentage of women in the case group appears lower because estimates are representative of the larger post‐9/11 veteran population, which is approximately 83% male. Race categories were not mutually exclusive.

A comparison of measures from the survey data for individuals who completed the interview with those who did not, revealed that completers had significantly higher Eating Disorder Diagnostic Scale‐5 and Depression Anxiety Stress Scale‐21 (Lovibond and Lovibond 1995) scores and were significantly older than non‐completers (all *p* < 0.05). See Table S2.

### 2.2 Confirmed EDs and Diagnostic Status

Table 2 shows the frequencies and proportions of different ED diagnostic groups in the sample of confirmed cases. The greatest number of participants met criteria for Other Specified Feeding and Eating Disorder (OSFED), followed by equal numbers with BED and Unspecified Eating Disorder (UFED), followed by BN; with one person fulfilling criteria for AN. The UFED cases could not easily be characterized as falling within the 3 DSM 5 separately designated disorders or OSFED. They can best be described as having a mix of ED symptoms including a clinical level of concern about shape, weight eating and their control together with a mix of dietary restraint, out of control eating (not always objectively large), and the use of a variety of extreme forms of weight control (compensatory, non‐compensatory or both). For those with UFED, symptoms were present for the required 3 months, but the pattern was not consistent enough for any other OSFED diagnosis.

**Table 2:** Current ED diagnoses of the confirmed cases (*n* = 91).

	Women *N* (%)	Men *N* (%)	Other gender *N* (%)	Total *N*
Anorexia nervosa (AN)	1 (1.4)	0 (0.0)	0	1 (0.5)
Bulimia nervosa (BN)	8 (11.9)	3 (13.1)	0	11 (12.4)
Binge Eating Disorder (BED)	14 (19.5)	9 (32.9)	0	23 (27.3)
Other Specified Eating Disorder (OSFED)	23 (42.7)	9 (32.7)	1 (34.4)	33 (36.4)
*Atypical AN*	4 (9.1)	1 (3.4)	1 (34.4)	6 (6.1)
*Subthreshold BN*	6 (13.3)	1 (4.0)	0 (0.0)	7 (7.4)
*Subthreshold BED*	10 (16.2)	3 (4.2)	0 (0.0)	13 (8.6)
*Purging Disorder*	2 (2.2)	3 (17.9)	0 (0.0)	5 (11.8)
*Night Eating Syndrome*	1 (1.9)	1 (3.2)	0 (0.0)	2 (2.7)
Other Unspecified Eating Disorder (UFED)	16 (24.7)	6 (21.2)	1 (65.6)	23 (23.4)

*Note:* Raw frequencies and weighted percentages are reported.

Of the potential controls excluded, one met the criteria for BN, 2 for OSFED (subthreshold BED and NES) and 6 for UFED. Weighted prevalence estimates for confirmed EDs among both potential cases and controls interviewed in the sample of 1494 were as follows: AN (0.01%, SE = 0.0001), BN (0.6%, SE = 0.003), BED (1.4%, SE = 0.004), OSFED (1.6%, SE = 0.004), UFED (1.6%, SE = 0.005). Prevalence for any ED (including UFED) was 5.2% (SE = 0.01) with prevalence for any specified disorder (AN, BN, BED or OSFED) being 3.6% (SE = 0.01).

### 2.3 Comorbid Psychiatric Disorder at Interview

Seventy‐nine of the 91 (86.8% unweighted) confirmed cases had a diagnosis of at least one other co‐morbid psychiatric disorder, with many having more than one (see Table 3).

**Table 3:** Comorbid psychiatric disorders in the sample of cases.

	Women *N* (weighted %)	Men *N* (weighted %)	Other gender *N* (weighted %)	Total *N* (weighted %)
Major depressive episode (lifetime)	45 (73.7)	15 (55.7)	1 (65.6)	61 (62.6)
Current	29 (46.4)	12 (47.7)	1 (65.6)	42 (47.5)
Past	16 (27.3)	3 (8.0)	0 (0.0)	19 (15.1)
Panic disorder (lifetime)	21 (30.4)	7 (26.8)	1 (65.6)	29 (28.9)
Current	15 (21.3)	5 (21.8)	1 (65.6)	21 (22.5)
Past	6 (9.1)	2 (5.0)	0 (0.0)	8 (6.4)
Social anxiety (lifetime)	25 (32.5)	9 (33.6)	2 (100.0)	36 (34.4)
Current	25 (32.5)	8 (31.9)	2 (100.0)	35 (33.4)
Past	0 (0.00)	1 (1.7)	0 (0.0)	1 (1.0)
Generalized anxiety disorder (lifetime)	33 (57.2)	13 (47.9)	2 (100.0)	48 (52.3)
Current	29 (50.2)	12 (45.2)	2 (100.0)	43 (48.1)
Past	4 (7.0)	1 (2.7)	0 (0.0)	5 (4.2)
Agoraphobia (lifetime)	14 (16.7)	6 (21.2)	1 (34.4)	21 (19.8)
Current	14 (16.7)	6 (21.2)	1 (34.4)	21 (19.8)
Past	0 (0.0)	0 (0.0)	0 (0.0)	0 (0.0)
Obsessional Compulsive Disorder (lifetime)	12 (17.9)	6 (22.7)	0 (0.0)	18 (20.5)
Current	12 (17.9)	4 (16.5)	0 (0.0)	16 (16.7)
Past	0 (0.0)	2 (6.2)	0 (0.0)	2 (3.8)
PTSD (lifetime)	37 (57.2)	16 (53.7)	2 (100.0)	55 (55.9)
Current	25 (38.4)	14 (42.5)	2 (100.0)	41 (42.1)
Past	12 (18.7)	2 (11.2)	0 (0.0)	14 (13.8)
Alcohol use disorder (lifetime)	10 (12.7)	6 (20.4)	0 (0.0)	16 (17.1)
Current	5 (5.2)	5 (18.0)	0 (0.0)	10 (12.9)
Past	5 (7.6)	1 (2.4)	0 (0.0)	6 (4.3)
Substance use disorder (any; lifetime)	2 (3.6)	3 (9.0)	0 (0.0)	5 (6.8)
Current	1 (2.1)	2 (5.0)	0 (0.0)	3 (3.8)
Past	1 (4.0)	1 (3.3)	0 (0.0)	2 (3.0)

*Note:* Substance use disorders included sedative, hypnotic or anxiolytic; cannabis; stimulant; opioid; inhalant; phencyclidine; hallucinogen; or other substance use. Raw frequencies and weighted percentages are reported.

### 2.4 Risk Factors

Most cases had an index age before joining the military (see Table 1). A minority (*n* = 20) had an index age after joining, 19 of whom completed the RFI. Further details of risk factor exposure (before index age) of those whose index age was before and after joining the military is provided in Table S3.

As can be seen from the exploratory analysis (Table 4), military risk factors before index age were not significantly associated with an increased risk of disorder. Within each of the other previously established family and individual risk domains and other environmental circumstances at least some, if not all items, were associated with an increased risk of an eating disorder.

**Table 4:** Risk factor exposure before index age (adjusted on age and sex, and accounting for sample weights).

Risk factors and domains	Cases (*n* = 87)	Controls (*n* = 45)	Odds ratio (95% CI)	*p*‐value
Parenting quality				
Little affection from parent/caregiver	45	11	2.77 (0.93, 8.31	0.7
Parent/caregiver criticism	52	11	561 (1.83, 17.24)	0.01
Parent/caregiver high demands	48	10	8.04 (2.44, 26.49)	0.01
Parent caregiver disruptions (any; death, absence, separation, illness, change of caregiver)	60	27	1.01 (0.34 3.2)	0.98
Family eating and weight concerns				
Household restrictive diet (for religious, health, or weight control reasons)	13	6	0.62 (0.16, 2.47)	0.50
Household eating concerns (any; dieting, overeating, restrictive diet, chaotic meals, meal tension (food related), negative comments about appearance, negative comments about eating)	79	27	7.56 (1.85, 30.90)	0.01
Individual and family health				
Childhood obesity	19	4	6.71 (1.45, 31.02)	0.02
Family history of obesity	70	22	4.58 (1.50, 13.96)	0.01
Family mental health				
Family history of any psychiatric disorder	55	12	5.39 (1.82, 15.99)	≤ 0.01
Individual mental health				
Individual mental health vulnerability, any (conduct, absenteeism, school anxiety, no friends, negative self‐evaluation, shyness, difficulty expressing emotions, perfectionism, conscientiousness)	83	30	8.30 (2.04 33.78)	≤ 0.01
Military risk factors				
Military sexual trauma, any	3	2	0.39 (0.07, 2.28)	0.30
Military stressful events (combat and other adverse events)	5	2	1.83 (0.28, 10.46)	0.53
Military weight issues (negative comments about appearance or eating, advised to lose weight, diet prescribed)	4	2	1.65 (0.22, 12.13)	0.62
Abuse				
Physical abuse	58	14	2.98 (1.07, 8.34)	0.04
Abuse (bullying, teasing, discrimination), any	55	14	4.06 (1.43, 11.59)	0.01
Sexual abuse (nonmilitary), any	40	7	4.96 (1.53, 16.13)	0.01
Other environmental circumstances				
Number of life events^a^ in 12 months before index age (mean)	3.6	1.8	1.30 (0.99, 1.71)	0.06
Food deprivation/insecurity	21	1	30.75 (3.22, 293.99)	≤ 0.01

*Note:* Family history of ED: model fitting questionable due to zero cell, and results are not presented here. Models controlled for gender.

^a^ Life events assessed by RFI in year before index age: Moving house; moving to a new country; personal illness; pregnancy/birth of a child; death of someone close; illness of someone close; anyone left/joined family; breakup of significant relationship; sexually abused; physically assaulted; exam/school pressure; other stress/pressure; subject to critical comments about weight and shape; personal safety threatened; felt unsafe at home; unsafe in neighborhood; unsafe at school/work; anything else significant.

## 3 Discussion

We employed a gold‐standard two stage design (Lindvall Dahlgren and Wisting 2016) in a nationally representative sample of veteran women and men who had recently separated from military service to diagnose EDs by interview. Probable cases and those without an ED were initially identified by screening questionnaires and then interviewed to establish case status and confirm probable comorbid psychiatric diagnoses. In an exploratory case‐control investigation, the occurrence before disorder onset of previously established risk factors and potential additional military risk factors was investigated.

As anticipated, the weighted prevalence estimate for any ED of 5.2%, established at interview, was lower than the weighted estimate (7.9%) for probable cases identified by survey in this sample (Mitchell et al. 2022). It was also much lower than the unweighted estimates reported in a recent self‐report study (Masheb et al. 2021). Of the probable cases identified, 77% of those interviewed met criteria for any ED, and 58% met criteria for any DSM‐5 specified disorder (AN, BN, BED and OSFED). Prevalence estimates of EDs established by interview are lower than estimates from self‐report surveys (Galmiche et al. 2019; Lindvall Dahlgren and Wisting 2016), likely because not all respondents interpret complex behavior and attitudes in a uniform way. For example, assessing binge eating by self‐report can be challenging (Fairburn and Beglin 1994). It is also possible that some respondents may report behavior and attitudes that they would not disclose to an interviewer in the more anonymous format of a questionnaire (Masheb et al. 2021).

The breakdown of the various diagnostic groups with roughly equal proportions of those meeting criteria for the three specific DSM‐5 diagnoses and for OSFED was as expected from previous literature (Galmiche et al. 2019). Rates of atypical anorexia nervosa (AAN) were much lower than those reported in a recent prevalence study of EDs in veterans assessed by self‐report (Masheb et al. 2021). Differences are likely to be attributable to both the method of assessment and different definitions of AAN. As Walsh and colleagues (Walsh, Hagan, and Lockwood 2023) have noted, there is currently no agreement on the exact time frame or amount of weight loss required for this diagnosis, nor has there been consensus on its precise relationship to other ED diagnostic categories. In our study, we used a 3‐month time frame, as we did for all other diagnoses.

Of note, we found that nearly a third of the cases we identified did not clearly fall into one of the OSFED categories and presented with mixed symptoms that could not easily be classified within current schemes except as UFED. This suggests that, with expanding knowledge about the diversity of ED presentations, we might need to think further about how these disorders are best classified.

As expected, we found high rates of comorbid disorders in our sample with lifetime rates of anxiety and depression similar to those reported in a nationally representative sample of US adults with ED (Udo and Grilo 2019), while rates of PTSD (55.9%) were higher. Given that the literature suggests that those with comorbid psychiatric disorders (primarily anxiety and depression), as compared with those without these conditions, achieve less good outcomes with existing treatments (Lydecker and Grilo 2022), it may not only be important that this group receive timely treatment, but also that we develop treatment approaches that are better able to meet their needs (Wade, Shafran, and Cooper 2024). The occurrence of PTSD may require special attention as the symptoms of PTSD and ED may interact, with the two disorders reciprocally maintaining each other and thereby making treatment especially challenging (Mitchell et al. 2021; Trottier et al. 2022).

The risk factors that we identified in our exploratory analyses were similar to those previously identified in the literature (Hilbert et al. 2014; Striegel‐Moore et al. 2002, 2005). We found preliminary evidence for previously identified general risk factors for ED such as conduct disorder, family psychiatric disorder, bullying, physical and sexual abuse as well as ED specific risk factors such as individual and family history of obesity, and high parental demands. Ideally, as in some previous risk factor studies (Gonçalves et al. 2016; Machado et al. 2014; Striegel‐Moore et al. 2005) we would have had two control groups: with no ED and no psychiatric disorder and another with no ED but other psychiatric disorder. This would have allowed us to distinguish in this study between general risk factors for ED and those specific only to ED. Since budgetary and other practicalities made this impossible, we chose to have a control group with no psychiatric disorder following other published studies (Hilbert et al. 2014; Pike et al. 2021). Military factors thought to be potential risk factors for the occurrence of EDs in veterans were not confirmed as such. The likely reason for this is that we were assessing risk factors before the onset of disorder and, in this sample, the mean index age, defined as the first onset of any persisting ED symptom or behavior, was 17.2. The number of people for whom index age was sufficiently late for it to be possible for military risk factors to have occurred before onset was relatively small and it is therefore difficult to interpret these data. This finding was not anticipated. A much larger interview sample might have produced greater numbers of those with later onset of disorder to allow an examination of military risk factorsfor ED onset using a case control design. Although military risk factors were not shown to play a role pre‐onset in the present study, they might well have contributed to the persistence or worsening of any pre‐existing disorder. This would need to be examined in a longitudinal study.

This study has several limitations. Sample sizes were small. We had difficulty recruiting potential case and control participants as planned despite telephone calls, text messages and letters. This was especially so for control participants, and we were relatively less successful in recruiting women as matches for cases as opposed to men. Our difficulties were possibly compounded by recruiting during COVID‐19 lockdown periods. Some potential participants were likely lost due to illness and women may have been disproportionately impacted by school shutdowns making participation difficult. Another potential difficulty was the delay between indicating a willingness to be contacted for interview at the time of the survey and subsequently being contacted. There were delays in commencing the interview part of the study as sections of the protocol had to be revised to make the study fully remote during COVID‐19 shutdowns and obtain further IRB approval. It is difficult to be sure why we were unable to make initial contact with many participants. In some cases, we no longer had correct contact details, but, in others, it is likely that they were unwilling to participate. Caution is particularly required in interpreting the risk factor findings as the case control comparisons were based on small numbers with consequent wide confidence limits for our estimates.

Survey data were used to identify potential cases and controls. Given that some participants were interviewed as long as 18 months after completing the survey, it is possible that the diagnostic status of participants changed in the interim, with some cases no longer meeting criteria for an ED at interview, and some controls developing an ED before the interview. However, in both circumstances these explanations seem unlikely. Without treatment, EDs tend to persist and very few those surveyed reported receiving treatment (Mitchell et al. 2022). All potential controls not subsequently confirmed as controls had an identified index age for the onset of disorder well before they completed the survey. While it cannot be established that the disorder persisted without periods of remission, this again seems unlikely given what is known about the persistence of disorder without treatment.

Prevalence estimates can be difficult to establish. In this study, as is the case for many such studies, response rates were relatively low (Galmiche et al. 2019). The survey response rate was 19.4%, a rate typical of military and veteran surveys (Coughlin et al. 2011). We were only able to interview 60% of the identified probable cases. These response rate problems were mitigated to some extent by using sample weights to address nonresponse and increase the representativeness of the sample. Rates may be higher if those with disorder are less likely to consent to being interviewed and if screening tools lack adequate sensitivity to identify all probable cases.

Strengths of the study include a nationally representative sample of veteran women and men, the use of well‐established interviews to determine ED and psychiatric diagnosis and weighting to reduce the problems of nonresponse.

In recently separated veterans, we found similar rates of ED and co‐occurring psychiatric disorder, when diagnosed at interview, as reported for nonveteran populations, although rates of PTSD were higher. Given that co‐occurring psychiatric disorders, especially PTSD, may complicate achieving good treatment outcomes with existing evidence‐based treatments, there is a need to adapt and subsequently test more personalized approaches for this group. Improving our understanding of maintaining and exacerbating factors, including military factors, is likely necessary to improve treatments for this population.

## Author Contributions

**Zafra Cooper:** conceptualization, funding acquisition, supervision, methodology, writing–original draft and review and editing. **Brian N. Smith:** writing–review and editing. **Shannon Kehle‐Forbes:** writing–review and editing. **Julianne Dorset:** investigation, administration, writing–review and editing. **Karen S. Mitchell:** conceptualization, funding acquisition, formal analysis, writing–original draft, review and editing.

## Conflicts of Interest

The authors declare no conflicts of interest.

## Supporting information

Supporting information.

## Data Availability

Research data shared on reasonable request and with a data sharing agreement.
