# Supplementary material for: Eating Disorders, Co‐Morbid Disorders and Early Risk Factors Amongst Post‐9/11 Veteran Men and Women
Source: J Clin Psychol. 2024 Dec 8;81(3):133–44. doi: 10.1002/jclp.23756 (PMC11802485; doi:10.1002/jclp.23756)
Supplement: Supplementary file 1 — Supporting information. [file JCLP-81-133-s001.docx]

**Supporting Information - Selection of case and control participants and comparison of interview completers and non- completers**

**Selection of Potential Cases and Controls**

Responses from the survey data were used to select potential cases and controls for the interview portion of the study. Survey participants were selected for the potential case group if they endorsed specific behaviors needed for an ED diagnosis on any one of the ED measures listed below and exceeded the cutoff on the CIA. At the time of initiating the study there was no ideal screening measure available, especially not for the veteran population. While the EDDS-5 had the advantage of a three-month time frame, there was virtually no validation or psychometric data available. The EDE-Q had the disadvantage of only a one-month time frame. Although there was a wealth of validation and other psychometric data for the EDE-Q, none was with a veteran population. We decided to maximize the information available to us and potentially use both together with the Clinical impairment assessment (CIA) to avoid over selecting potential cases. In the event, a mistake was made on the survey: to reduce participant burden those who did not endorse binge eating were not asked about extreme methods of weight control. Hence compensatory behaviors and purging could not be assessed using the EDDS. Therefore, we used binge eating from the EDDS to get the 3-month time frame and used the EDE-Q to assess purging.

Participants in the potential control group could not meet cutoffs on any ED measure or any of the five measures of comorbid mental health conditions (see Table S1). Potential cases were administered SCID modules to assess comorbid conditions based on whether they exceeded cutoffs on the additional mental health measures.

Cutoff scores were based on the best available guidelines from the literature at the time the study was initiated for the EDE-Q (Lavender et al., 2010; Mond et al., 2006), SCOFF (Luck et al., 2002), CIA (Bohn et al., 2008), PHQ-9 (Kroenke et al., 2001), DASS-21 (Henry & Crawford, 2005; Mitchell et al., 2008), PCL-5 (Blevins et al., 2015), AUDIT (Babor et al., 2001), and DUDIT (Hildebrand, 2015). Please note that a subsequent publication has suggested a lower CIA cut off may be more appropriate for men with potential eating disorders (Richson et al. 2021). These data were not available at the time cases and controls were identified from the survey study data, nor were they at the time interviewing for the study began.

*Table S1.* Cutoff scores on ED measures and comorbid mental health disorder measures

| **Measure** | **Cutoff for ED group** | | **Cutoff for Control Group** | |
| --- | --- | --- | --- | --- |
|  | **Women** | **Men** | **Women** | **Men** |
| **Eating Disorder Measures** |  |  |  |  |
| EDDS-5 | Binge eating=yes  **AND**  CIA > 16 | Binge eating=yes  **AND**  CIA > 16 | #4=no | #4=no |
| EDE-Q | Self-induced vomiting > 4 **OR** laxative use > 4 **OR** excessive exercise > 4 **OR** fasting > 1  **AND** CIA > 16 | Self-induced vomiting > 4 **OR** laxative use > 4 **OR** excessive exercise > 4 **OR** fasting > 1  **AND** CIA > 16 | EDE-Q global score < 2.8  **AND**  Self-induced vomiting = 0 **AND** laxative use = 0 **AND** excessive exercise < 1 **AND** fasting = 0 | EDE-Q global score < 1.8  **AND**  Self-induced vomiting = 0 **AND** laxative use = 0 **AND** excessive exercise < 1 **AND** fasting = 0 |
| SCOFF | -- | -- | < 2 | < 2 |
| CIA | > 16 (in conjunction with EDDS or EDE-Q responses) | N/A | < 16 | < 16 |
| **Comorbid Conditions** |  |  |  |  |
| PHQ-9 | -- | -- | < 15 | < 15 |
| DASS-21 | -- | -- | < 8 (moderate) | < 8 (moderate) |
| PCL-5 | -- | -- | < 33 | < 33 |
| AUDIT | -- | -- | < 6 | < 8 |
| DUDIT | -- | -- | 0 | < 3 |

Note: EDDS-5=Eating Disorder Diagnostic Scale, Eating Disorder Examination Questionnaire=EDE-Q, CIA= Clinical Impairment Assessment, PHQ-9= Patient Health Questionnaire-9, DASS-21=Depression Anxiety Stress Scales-21, PTSD Checklist-5=PCL-5, AUDIT=Alcohol Use Disorder Identification Test, DUDIT=Drug Use Disorder Identification Test. Cutoff scores on the DASS-21 are for each individual subscale (depression, anxiety, stress).

*Table S2.* Comparison of interview completers and non-completers

|  | Completer  (Mean) | Non-completer  (Mean) | B | SE | p | OR  (95% CI) |
| --- | --- | --- | --- | --- | --- | --- |
| EDDS-5 | 5.05 | -3.35 | 0.04 | 0.01 | *p* < .001 | 1.05  (1.02, 1.07) |
| PCL-5 | 14.84 | 11.76 | 0.01 | 0.01 | 0.44 | 1.01  (0.99, 1.02) |
| DASS-Depression | 13.48 | 6.35 | 0.04 | 0.01 | <0.001 | 1.04  (1.02, 1.07) |
| DASS-Anxiety | 9.98 | 4.64 | 0.05 | 0.01 | <0.01 | 1.05  (1.02, 1.07) |
| DASS-Stress | 14.73 | 6.36 | 0.05 | 0.01 | *p* < .001 | 1.05  (1.03, 1.08) |
| AUDIT | 3.96 | 3.01 | 0.05 | 0.03 | 0.14 | 1.05  (0.98, 1.12) |
| DUDIT | 0.53 | 0.61 | -0.01 | 0.03 | 0.73 | 0.99  (0.93, 1.05) |
| Age | 28.87 | 26.48 | 0.07 | 0.02 | <0.01 | 1.08  (1.03, 1.13) |
|  | Completer  (%) | Non-completer  (%) | B | SE | p | OR  (95% CI) |
| Female gender | 21.62 | 24.50 | -0.16 | 0.25 | 0.52 | 0.85  (0.53, 1.38) |
| Latinx/Hispanic | 12.83 | 15.45 | -0.22 | 0.37 | 0.56 | 0.81  (0.39, 1.67) |
| Pacific Islander or Native Hawaiian | 0.00 | 0.52 | -13.76 | 0.88 | <0.001 | 1.06e-06  (1.90e-07, 5.95e-06) |
| American Indian or Alaska Native | 1.15 | 6.13 | -1.58 | 0.70 | 0.02 | 0.21  (0.05, 0.81) |
| Black | 12.70 | 21.51 | -0.50 | 0.64 | 0.44 | 0.61  (0.17, 2.12) |
| White | 79.84 | 70.06 | 0.11 | 0.64 | 0.87 | 1.11  (0.32, 3.88) |
| Asian | 6.98 | 6.24 | -0.17 | 0.81 | 0.83 | 0.84  (0.17, 4.06) |
| Other race | 4.25 | 3.27 | 0.32 | 0.82 | 0.70 | 1.37  (0.27, 6.93) |

Note: Independent variables were assessed during the survey data collection phase of the study. EDDS-5=Eating Disorder Diagnostic Scale-5, PCL-5=PTSD Checklist-5, DASS=Depression Anxiety Stress Scales (21 item version), AUDIT=Alcohol Use Disorders Identification Test, DUDIT=Drug Use Disorders Identification Test, SE=standard error, OR=odds ratio, CI=confidence interval. The outcome was coded as interview completer=1 and non-completer=0. Separate models were estimated for each independent variable, with the exception of race categories, which were included in one model. Race categories were not mutually exclusive.

*Table S3.*  Risk factor exposure before index age

| **Risk Factors and Domains** | **Early Onset Cases (n=65)** | **Late Onset Cases (n=19)** |
| --- | --- | --- |
| PARENTING QUALITY |  |  |
| Little affection from parent/caregiver | 31 (47.7%) | 9 (45.0%) |
| Parent/caregiver criticism | 35 (53.8%) | 12 (60.0%) |
| Parent/caregiver high demands | 34 (52.3%) | 12 (60.0%) |
| Parent caregiver disruptions (any; death, absence, separation, illness, change of caregiver) | 38 (58.5%) | 16 (80.0%) |
| FAMILY EATING AND WEIGHT CONCERNS |  |  |
| Household restrictive diet (for religious, health, or weight control reasons) | 9 (13.8%) | 2 (10.0%) |
| Household eating concerns (any; dieting, overeating, restrictive diet, chaotic meals, meal tension (food related), negative comments about appearance, negative comments about eating) | 55 (84.6%) | 18 (90.0%) |
| INDIVIDUAL AND FAMILY HEALTH |  |  |
| Childhood obesity | 15 (23.1%) | 3 (15.0%) |
| Family history of obesity | 49 (75.4%) | 15 (75.0%) |
| FAMILY MENTAL HEALTH |  |  |
| Family history of any psychiatric disorder | 39 (60.0%) | 11 (55.0%) |
| INDIVIDUAL MENTAL HEALTH |  |  |
| Individual mental health vulnerability, any (conduct, absenteeism, school anxiety, no friends, negative self-evaluation, shyness, difficulty expressing emotions, perfectionism, conscientiousness) | 58 (89.2%) | 1 (5.0%) |
| MILITARY RISK FACTORS |  |  |
| Military sexual trauma, any | 0 | 3 (15.0%) |
| Military stressful events  (combat and other adverse events) | 0 | 4 (20.0%) |
| Military weight issues (negative comments about appearance or eating, advised to lose weight, diet prescribed) | 0 | 3 (15.0%) |
| ABUSE |  |  |
| Physical abuse | 39 (60.0%) | 13 (65.0%) |
| Abuse (bullying, teasing, discrimination), any | 39 (60.0%) | 11 (55.0%) |
| Sexual abuse (non-military), any | 24 (36.9%) | 11 (55.0%) |
| OTHER ENVIRONMENTAL CIRCUMSTANCES |  |  |
| Number of life events^a^  **in 12 months prior to index age (mean)** | 3.3 (2.4) | 4.5 (2.3) |
| Food deprivation/insecurity | 12 (18.5%) | 5 (25.0%) |

Notes: Early onset cases reported an age of onset prior to entering the military. Late onset cases reported an age of onset after entering the military; 6 participants had an age of onset around the time of military enlistment and are not included here. One participant in the late onset group did not complete the RFI.

References

Babor, T. F., Higgins-Biddle, J. C., Saunders, J. B., & & Monteiro, M. G. (2001). *The Alcohol Use Disorders Identification Test: Guidelines for use in primary care*.

Blevins, C. A., Weathers, F. W., Davis, M. T., Witte, T. K., & Domino, J. L. (2015). The Posttraumatic Stress Disorder Checklist for DSM-5 (PCL-5): Development and initial psychometric evaluation. *Journal of Traumatic Stress*, *28*(6), 489–498. https://doi.org/10.1002/jts.22059

Bohn, K., Doll, H. A., Cooper, Z., O’Connor, M., Palmer, R. L., & Fairburn, C. G. (2008). The measurement of impairment due to eating disorder psychopathology. *Behaviour Research and Therapy*, *46*(10), 1105–1110. https://doi.org/10.1016/j.brat.2008.06.012

Henry, J. D., & Crawford, J. R. (2005). The short-form version of the Depression Anxiety Stress Scales (DASS-21): Construct validity and normative data in a large non-clinical sample. *The British Journal of Clinical Psychology*, *44*(Pt 2), 227–239. https://doi.org/10.1348/014466505X29657

Hildebrand, M. (2015). The psychometric properties of the Drug Use Disorders Identification Test (DUDIT): A review of recent research. *Journal of Substance Abuse Treatment*, *53*, 52–59. https://doi.org/10.1016/j.jsat.2015.01.008

Kroenke, K., Spitzer, R. L., & Williams, J. B. (2001). The PHQ-9: Validity of a brief depression severity measure. *Journal of General Internal Medicine*, *16*(9), 606–613.

Lavender, J. M., De Young, K. P., & Anderson, D. A. (2010). Eating Disorder Examination Questionnaire (EDE-Q): Norms for undergraduate men. *Eating Behaviors*, *11*(2), 119–121. https://doi.org/10.1016/j.eatbeh.2009.09.005

Luck, A. J., Morgan, J. F., Reid, F., O’Brien, A., Brunton, J., Price, C., Perry, L., & Lacey, J. H. (2002). The SCOFF questionnaire and clinical interview for eating disorders in general practice: Comparative study. *BMJ : British Medical Journal*, *325*(7367), 755–756.

Mitchell, M. C., Burns, N. R., & Dorstyn, D. S. (2008). Screening for depression and anxiety in spinal cord injury with DASS-21. *Spinal Cord*, *46*(8), 547–551. https://doi.org/10.1038/sj.sc.3102154

Mond, J. M., Hay, P. J., Rodgers, B., & Owen, C. (2006). Eating Disorder Examination Questionnaire (EDE-Q): Norms for young adult women. *Behaviour Research and Therapy*, *44*, 53.

Richson, B. N., Johnson, S. N., Swanson, T. J., Christensen, K. A., Forbush, K. T., & Wildes, J. E. (2021). Predicting probable eating disorder case-status in men using the Clinical Impairment Assessment: Evidence for a gender-specific threshold. *Eating Behaviors*, *42*, 101541. https://doi.org/10.1016/j.eatbeh.2021.101541
